# Pathogen Profile of Pediatric Community‐Acquired Pneumonia in Wuhan, 2023: A BALF‐Based Study

**DOI:** 10.1155/carj/8872950

**Published:** 2026-07-10

**Authors:** Zhongjian Wang, Yi Gan, Miao Wang, Yunyang Hu, Yabin Wu, Ying Cheng, Tao Huang, Danna Tu, Junhua Shu, Xiaoqin Zhou

**Affiliations:** ^1^ Department of Pediatrics, Maternal and Child Health Hospital of Hubei Province, Tongji Medical College, Huazhong University of Science and Technology, Wuhan, China, hust.edu.cn

**Keywords:** bronchoalveolar lavage fluid, children, community-acquired pneumonia, *Mycoplasma pneumoniae*, pathogen

## Abstract

**Objective:**

To analyze the distribution characteristics of pathogens in the bronchoalveolar lavage fluid of children with community‐acquired pneumonia.

**Methods:**

We retrospectively analyzed the etiological data of 1526 children diagnosed and treated at the Maternal and Child Health Hospital of Hubei Province from January 1, 2023, to December 31, 2023.

**Results:**

Pathogens were detected in 1524 of the 1526 children (99.9%). The five most frequently identified pathogens were *Mycoplasma pneumoniae*, rhinovirus, Epstein‒Barr virus, *Streptococcus pneumoniae*, and human bocavirus. The overall mixed infection rate was 62%. Epstein‒Barr virus, rhinovirus, *Streptococcus pneumoniae*, human bocavirus, and *Haemophilus influenzae* were the most common co‐infections of *Mycoplasma pneumoniae*. *Staphylococcus aureus* and *Pneumocystis jirovecii* infections were more common in males than in females. Human bocavirus, parainfluenza virus, respiratory syncytial virus, *Cytomegalovirus*, *Pneumocystis jirovecii*, and WU polyomavirus were more prevalent in infants and young children, and Epstein‒Barr virus was more common in preschool‐aged and school‐aged children. More than half (51.6%) of the *Mycoplasma pneumoniae* infections occurred in autumn. *Mycobacterium tuberculosis complex*, human parainfluenza virus, and *Cytomegalovirus* were more common in spring and summer, and influenza B virus and adenovirus were more common in autumn and winter. Respiratory syncytial virus, influenza A virus, and *Fusobacterium nucleatum* were more common in winter and spring. Enteroviruses were more prevalent in summer and autumn.

**Conclusion:**

The positive rate of pathogen detection in bronchoalveolar lavage fluid was extremely high. Most patients presented with mixed infections, and the distribution of some pathogens varied by sex, age, and season.

## 1. Introduction

Pneumonia is one of the leading causes of death in children under 5 years of age, and the vast majority of these cases are community‐acquired pneumonia (CAP) [1]. According to statistics, lower respiratory tract infections caused 652,572 deaths in children under 5 years of age worldwide in 2016 [2]. Following the COVID‐19 outbreak, pneumonia cases surged, creating a substantial global disease burden [3]. To control the pandemic, a series of nonpharmaceutical interventions (NPIs)—including social distancing, school closures, mask‐wearing, travel restrictions, and improved personal hygiene—were implemented worldwide, significantly reducing the transmission of 2019‐nCoV [4]. While these NPIs are effective in controlling the transmission of 2019‐nCoV, they also affect the seasonal transmission patterns of other respiratory viruses, such as the influenza virus, respiratory syncytial virus (RSV), human rhinovirus (HRV), human metapneumovirus (HMPV), and adenovirus (ADV) [5–7].

The change in the pathogen spectrum of respiratory tract infections in children after the outbreak of the 2019 novel coronavirus has attracted much attention. Liu et al. studied 7107 patients with lower respiratory tract infections. Compared with those in 2019, the infection rates of RSV, ADV, HMPV and influenza viruses decreased significantly in 2020, whereas HRV infection rates increased significantly. After September 2020, human parainfluenza virus (HPIV) infection resumed. In addition, there were significantly fewer co‐infections in 2020 than in 2019 [6]. Principi et al. suggested that postepidemic pathogen prevalence results from the interplay of various factors—including pathogen variation, host immunity, and mutual pathogen interference—each capable of altering the traditional epidemiology of a single virus [7]. Strengthening the surveillance of pathogen prevalence and vaccination is recommended.

Bronchoalveolar lavage fluid (BALF) is directly collected from the lesion site. Compared to blood or sputum culture, BALF more accurately reflects pulmonary etiology and guides antibiotic use [8–10]. To better understand the changes in the respiratory pathogen spectrum among children in the postpandemic era, we analyzed the distribution of BALF pathogens from children with CAP at the Maternal and Child Health Hospital of Hubei Province in 2023. This study aims to provide a basis for accurate diagnosis and rational drug use, as well as a scientific foundation for targeted prevention and control strategies.

## 2. Materials and Methods

### 2.1. Patients

The clinical data of 1526 children with CAP who underwent bronchoscopy and bronchoalveolar lavage at Maternal and Child Health Hospital of Hubei Province from January 1, 2023, to December 31, 2023, were retrospectively analyzed. The inclusion criteria were as follows: (1) had a clinical diagnosis of pediatric CAP, (2) were aged 28 days to 15 years, (3) had undergone bronchoscopy and bronchoalveolar lavage with the consent of their guardians, and (4) had complete clinical data. The exclusion criteria were as follows: (1) patients with contraindications to bronchoscopy, (2) patients with contraindications to anesthesia, (3) patients with serious underlying diseases, (4) patients whose guardians refused bronchoscopy, (5) patients with hospital‐acquired pneumonia, and (6) patients whose clinical data were incomplete. The indications for bronchoscopy in this study included the following: (1) recurrent or persistent wheezing; (2) unexplained chronic cough; (3) recurrent respiratory tract infection; and (4) abnormal chest imaging: (1) dysplasia and/or malformation of the trachea, bronchus, and lung; (2) atelectasis; (3) consolidation of the lung; (4) pulmonary mass lesions; (5) diffuse lung disease; and (6) etiological diagnosis and treatment of pulmonary infectious diseases. This study was approved by the hospital medical ethics committee (No. 2024‐107‐01).

### 2.2. Methods

Clinical information—including name, sex, age, imaging results, etiological findings, bronchoscopic findings, diagnosis, treatment, and complications—was collected via the hospital’s electronic medical record system. The contraindications for bronchoscopy included the following: (1) severe cardiopulmonary dysfunction; (2) severe arrhythmia; (3) persistent high fever; (4) active hemoptysis, severe hemorrhagic diseases, coagulopathy, or severe pulmonary hypertension; and (5) severe malnutrition in patients who could not tolerate surgery.

Reagents and instruments: Respiratory pathogenic microorganism multiple joint detection kit (Jinqi Rui Company, product number: KS608‐100HXD96), next‐generation sequencer (Jinqi Rui Company, product number: MiniSeqDx‐CN), etc.

Targeted high‐throughput next‐generation sequencing (tNGS): All patients underwent bronchoscopy, and BALF samples were collected. The collected samples were sent to Golden Med Medical Center for tNGS detection (multiplex PCR combined with next‐generation sequencing technology). After the BALF samples were thoroughly mixed, 1300 μL was centrifuged at 12,000 r/min for 5 min. After centrifugation, the supernatant was removed, 500 μL of the sample was retained, and the mixture was blown. A 500 μL sample was put into the bead mill tube of the extraction kit, and 50 μL of SDS was added to the wall‐breaking instrument. After the wall‐breaking treatment, the samples were centrifuged at 12,000 rpm for 5 min, and 400 μL of the supernatant was used for nucleic acid extraction. Ultramultiplex PCR was used to amplify and enrich the target sequence to form short fragments of nucleic acid without interruption. After purification, the products were added to the adapter, and a second round of PCR amplification was performed to construct the library. The library quality was controlled by an ultramicro spectrophotometer, and the libraries were sequenced. After the sequencing was completed, the data were analyzed to form the final report.

The sequencing depth of tNGS for samples in this study ranged from 500*x* to 1000*x*. Pathogen positivity was determined based on specific sequence counts and clinical context, with the following criteria: Definitive positive: ≥ 100 specific sequences from nonsingle‐target regions. Indeterminate (10–100 specific sequences): Requires verification via auxiliary methods (e.g., real‐time quantitative PCR) or repeat sampling. Low‐confidence (≤ 10 specific sequences): Requires repeat testing; a positive repeat result is prioritized for interpretation, while a negative repeat is classified as nonpositive. Adjusted thresholds: For certain viruses or bacteria, lower sequence count thresholds may apply, but only after comprehensive evaluation of clinical context and detection consistency. The detection range of tNGS in this study included 198 pathogens of the respiratory tract, including 80 bacteria, 79 viruses, 32 fungi, and seven other pathogens (including *Mycoplasma pneumoniae* [MP] and *Chlamydia pneumoniae*). The interpretation of tNGS results requires integrative analysis incorporating the pediatric patient’s clinical symptoms, laboratory test results, radiological findings, and treatment responses.

### 2.3. Statistical Analysis

SPSS 29 software was used; the count data are expressed as *n* (%), and the chi‐square test or Fisher’s exact probability test was used for comparisons between groups. *p* < 0.05 was considered statistically significant.

## 3. Results

### 3.1. Overall Distribution of Pathogens

**FIGURE 1:**
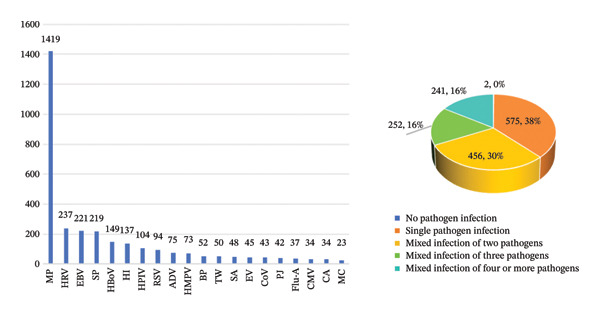
The number of patients infected with the top 20 common pathogens and the mixed infection status of different pathogens. Note: MP: *Mycoplasma pneumoniae*; HRV: human rhinovirus; EBV: Epstein–Barr virus; SP: *Streptococcus pneumoniae*; HBoV: human bocavirus; HI: *Haemophilus influenzae*; HPIV: human parainfluenza virus; RSV: respiratory syncytial virus; ADV: adenovirus; HMPV: human metapneumovirus; BP: *Bordetella pertussis*; TW: *Tropheryma whipplei*; SA: *Staphylococcus aureus*; EV: *Enterovirus*; CoV: coronavirus; PJ: *Pneumocystis jirovecii*; Flu‐A: influenza A virus; CMV: *Cytomegalovirus*; CA: *Candida albicans*; MC: *Moraxella catarrhalis*.

Pathogens were detected in 99.9% (1524/1526) of patients. The 20 most common pathogens are shown in Figure 1. Figure 1 illustrates the pathogen distribution characteristics via two key panels: The left panel displays the top 20 pathogens ranked by the number of positive patients, and the right panel uses a pie chart to depict the composition of single infections and mixed infections involving varying numbers of pathogens within the study cohort. The five most common pathogens were MP (93.0%, 1419/1526), HRV (15.5%, 237/1526), Epstein–Barr virus (EBV) (14.5%, 221/1526), *Streptococcus pneumoniae* (SP) (14.4%, 219/1526), and human bocavirus (HBoV) (9.8%, 149/1526). SP, *Haemophilus influenzae* (HI), *Bordetella pertussis* (BP), *Tropheryma whipplei* (TW) and *Staphylococcus aureus* (SA) were the five most common bacteria. The five most common viruses were HRV, EBV, HBoV, HPIV, and RSV. Of all patients, 575 (38%) had a single pathogen, 456 (30%) had two pathogens, 252 (16%) had three, and 241 (16%) had four or more (Figure 1), yielding a mixed infection rate of 62%. A high prevalence of co‐infections stands as a distinctive feature of this cohort, with MP‐containing combinations being the most frequently observed among co‐infection cases. The most common co‐infections were MP with EBV (13.6%, 208/1526), MP with HRV (13.5%, 206/1526), MP with SP (12.5%, 191/1526), MP with HBoV (8.9%, 136/1526), and MP with HI (7.4%, 113/1526).

### 3.2. Distribution of Pathogens by Sex

There were 745 males and 781 females. Table 1 shows the comparison of the distributions of some pathogens in different sexes. SA and *Pneumocystis jirovecii* (PJ) infections were more common in males than in females (*p* < 0.05). No significant sex differences were observed for other pathogens.

**TABLE 1:** Comparison of the distribution of some pathogens in different sexes (*n*, %).

Groups	MP	HRV	EBV	SP	HBoV	SA	PJ
Male (*n* = 745)	687 (92.2)	127 (17.0)	109 (14.6)	113 (15.2)	76 (10.2)	33 (4.4)	28 (3.8)
Female (*n* = 781)	732 (93.7)	110 (14.1)	112 (14.3)	106 (13.6)	73 (9.3)	15 (1.9)	14 (1.8)
*χ* ^2^	1.336	2.551	0.026	0.790	0.316	7.878	5.505
*p*	0.248	0.110	0.872	0.374	0.574	0.005	0.019

*Note:* HRV, human rhinovirus; HBoV, human bocavirus.

Abbreviations: EBV, Epstein–Barr virus; MP, *Mycoplasma pneumoniae*; PJ, *Pneumocystis jirovecii*; SA, *Staphylococcus aureus*; SP, *Streptococcus pneumoniae*.

### 3.3. Distribution of Pathogens by Age

**FIGURE 2:**
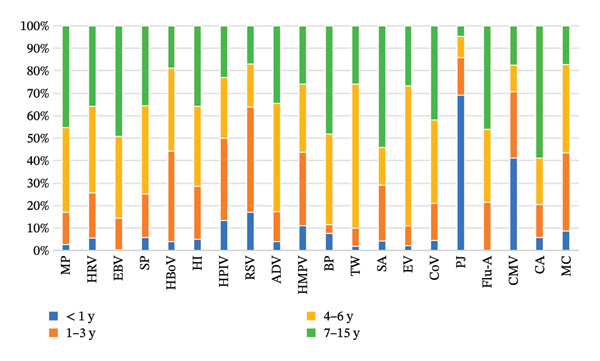
Distribution of the top 20 common pathogens in different age groups. Note: MP: *Mycoplasma pneumoniae*; HRV: human rhinovirus; EBV: Epstein–Barr virus; SP: *Streptococcus pneumoniae*; HBoV: human bocavirus; HI: *Haemophilus influenzae*; HPIV: human parainfluenza virus; RSV: respiratory syncytial virus; ADV: adenovirus; HMPV: human metapneumovirus; BP: *Bordetella pertussis*; TW: *Tropheryma whipplei*; SA: *Staphylococcus aureus*; EV: *Enterovirus;* CoV: coronavirus; PJ: *Pneumocystis jirovecii*; Flu‐A: influenza A virus; CMV: *Cytomegalovirus*; CA: *Candida albicans*; MC: *Moraxella catarrhalis*.

Patients were categorized into four age groups: infants (28 d–1 y, *n* = 56, 3.7%), toddlers (1–3 y, *n* = 239, 15.7%), preschool children (4–6 y, *n* = 564, 37%), and school‐aged children (7–15 y, *n* = 667, 43.7%). The distribution of the top 20 common pathogens in different age groups is shown in Figure 2. As can be observed from Figure 2, the detection rates of MP and EBV increase significantly with age; while pathogens such as RSV and *Cytomegalovirus* (CMV) show relatively higher detection rates in the infant and toddler groups. Table 2 shows the comparison of the distributions of some pathogens in different age groups. HBoV, HPIV, RSV, CMV, PJ and WU polyomavirus were more common in infants and young children (*p* < 0.05). EBV infection was more common in preschool‐aged and school‐aged children (*p* < 0.05).

**TABLE 2:** Comparison of the distribution of some pathogens in different age groups (*n*, %).

Groups	EBV	HBoV	HPIV	RSV	CMV	PJ	WUPyV
≤ 3 y (*n* = 295)	32 (10.8)	66 (22.4)	52 (17.6)	60 (20.3)	24 (8.1)	36 (12.2)	7 (2.4)
4–15 y (*n* = 1231)	189 (15.4)	83 (6.7)	52 (4.2)	34 (2.8)	10 (0.8)	6 (0.5)	3 (0.2)
*χ* ^2^	3.901	65.986	67.313	127.19	58.586	122.042	13.462
*p*	0.048	< 0.001	< 0.001	< 0.001	< 0.001	< 0.001	< 0.001

*Note:* HBoV, human bocavirus; HPIV, human parainfluenza virus; CMV, *Cytomegaloviru*s; WUPyV, WU polyomavirus.

Abbreviations: EBV, Epstein–Barr virus; PJ, *Pneumocystis jirovecii*; RSV, respiratory syncytial virus.

### 3.4. Distribution of Pathogens by Season

**FIGURE 3:**
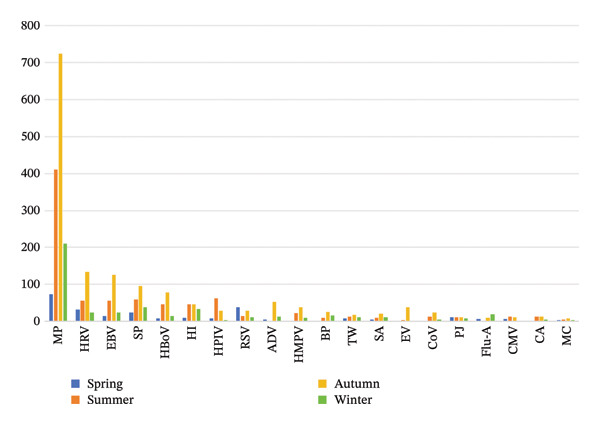
Distribution of the top 20 common pathogens in different seasons. Note: MP: *Mycoplasma pneumoniae*; HRV: human rhinovirus; EBV: Epstein–Barr virus; SP: *Streptococcus pneumoniae*; HBoV: human bocavirus; HI: *Haemophilus influenzae*; HPIV: human parainfluenza virus; RSV: respiratory syncytial virus; ADV: adenovirus; HMPV: human metapneumovirus; BP: *Bordetella pertussis*; TW: *Tropheryma whipplei*; SA: *Staphylococcus aureus*; EV: *Enterovirus*; CoV: coronavirus; PJ: *Pneumocystis jirovecii*; Flu‐A: influenza A virus; CMV: *Cytomegalovirus*; CA: *Candida albicans*; MC: *Moraxella catarrhalis*.

Patients were grouped by season: spring (March–May, *n* = 121, 7.9%), summer (June–August, *n* = 435, 28.5%), autumn (September–November, *n* = 732, 48%), and winter (December–February, *n* = 238, 15.6%). The distributions of the top 20 common pathogens in different seasons are shown in Figure 3. It can be clearly seen from Figure 3 that the detection rate of MP significantly increases in autumn. Meanwhile, HRV also shows a relatively high activity level in autumn. In contrast, some pathogens such as SP may not exhibit a very obvious seasonality. A comparison of the distributions of some pathogens in different seasons is shown in Tables 3 and 4. More than half (51.6%) of the MP infections occurred in autumn. *Mycobacterium tuberculosis complex* (MTBC), HPIV, and CMV infections were more common in spring and summer (*p* < 0.05). Influenza B virus (Flu‐B) and ADV infections were more common in autumn and winter (*p* < 0.05). RSV, influenza A virus (Flu‐A), and *Fusobacterium nucleatum* infections were more common in winter and spring (*p* < 0.05). *Enterovirus* infection was more common in summer and autumn (*p* < 0.05).

**TABLE 3:** Comparison of the distribution of some pathogens in spring/summer seasons and autumn/winter seasons (*n*, %).

Groups	MTBC	HPIV	CMV	Flu‐B	ADV
Spring + summer (*n* = 556)	8 (1.4)	71 (12.8)	21 (3.8)	0 (0)	8 (1.4)
Autumn + winter (*n* = 970)	1 (0.1)	33 (3.4)	13 (1.3)	14 (1.4)	67 (6.9)
*χ* ^2^	8.598	48.836	9.633	/	22.615
*p*	0.003	< 0.001	0.002	0.003	< 0.001

*Note:* MTBC, *Mycobacterium tuberculosis complex*; HPIV, human parainfluenza virus; CMV, *Cytomegaloviru*s; Flu‐B, influenza B virus; ADV, adenovirus.

**TABLE 4:** Comparison of the distribution of some pathogens in spring/winter seasons and summer/autumn seasons (*n*, %).

Groups	RSV	Flu‐A	FN	EV
Spring + winter (*n* = 359)	50 (13.9)	26 (7.2)	14 (3.9)	3 (0.8)
Summer + autumn (*n* = 1167)	44 (3.8)	11 (0.9)	8 (0.7)	42 (3.6)
*χ* ^2^	49.000	46.054	19.962	6.391
*p*	< 0.001	< 0.001	< 0.001	0.011

*Note:* Flu‐A, influenza A virus; EV, *Enterovirus*.

Abbreviations: FN, *Fusobacterium nucleatum*; RSV, respiratory syncytial virus.

## 4. Discussion

Pediatric CAP remains a significant cause of mortality. Rapid pathogen identification and appropriate anti‐infective therapy are crucial [2]. Alveolar biopsy tissue and BALF are the most accurate samples for CAP pathogen detection [11, 12]. In 2023, multiple pathogens were detected after the COVID‐19 epidemic. This study summarizes the distribution characteristics of the BALF pathogen spectrum in children with CAP in 2023.

In this study, the overall pathogen detection rate in BALF was 99.9%. Lv et al. reported a total positive rate of 77.87% among 4804 children with respiratory infections [13], while Wu et al. reported 67.5% among 10,435 children [14]. These differences are likely attributable to variations in region, host status, detection method sensitivity, and sample type. The five most common pathogens identified in this study were MP (93.0%), HRV, EBV, SP, and HBoV. Wang et al. reported that the pathogens most commonly detected in BALF samples from 573 children were ADV (21.82%), MP (20.24%), HRV (13.96%), SP (8.90%), and HI (8.90%) [15]. The significantly higher MP detection rate in our study is likely due to our selection of severe cases and the high sensitivity of tNGS for detecting MP nucleic acid in BALF. Most of the children in this study had atelectasis, pulmonary consolidation, or severe pneumonia. A multiregional outbreak of MP after the COVID‐19 pandemic is a well‐documented global phenomenon [16–18].

The most common bacteria in this study were SP and HI, consistent with other reports [2, 15]. The two most common viruses were HRV and EBV, which are different from the findings of other studies. Shi and Huang reported that HRV and RSV were the two most common viruses in a study of 10,396 children with acute respiratory tract infection from 2021 to 2022 [19]. The higher EBV detection rate in this study was considered to be related to immune abnormalities after the COVID‐19 pandemic. The mixed infection rate in our study was 62%, with the most common combinations being MP + EBV (13.6%), MP + HRV (13.5%), and MP + SP (12.5%). Zhao et al. performed pathogen detection on 4148 CAP children, 38.18% of whom had mixed infections, and the most common combination was MP combined with HRV [20]. The higher mixed infection rate in this study compared to other reports is primarily attributable to differences in sample types and subject selection, as well as post‐COVID‐19 immune dysregulation and the prolonged impact of NPIs. Of note, the detection of multiple pathogens by tNGS may represent co‐detection rather than true simultaneous pathogenic infection in all cases. Therefore, interpretation should follow the principle of integrating pathogen abundance, pathogenicity, and the patient’s clinical manifestations to determine clinical significance.

In this study, only SA and PJ infections showed a sex‐specific distribution, being more common in males. Zhang et al. reported that there was no statistically significant difference in the sex distribution of different pathogens during the COVID‐19 epidemic [21]. Li et al. reported that there was no sex difference in the pathogen detection rate; however, women were more likely to be infected with viruses, whereas men were more likely to be infected with SP [22]. A study from India revealed that the incidence of acute lower respiratory infections in children under 5 years of age was greater in boys than in girls, and boys had a 2.4‐fold higher rate of acute respiratory infection–related hospitalizations [23]. Zhu et al. reported that *Klebsiella pneumoniae*, *Acinetobacter baumannii*, *Pseudomonas aeruginosa*, *Stenotrophomonas maltophilia*, and *Candida albicans* were more common in male infants. HI, *Moraxella catarrhalis*, and SP were more common in female infants [24]. These data suggest sex‐specific differences exist for certain pathogens.

Pathogen distribution is also associated with age. In our study, HBoV, HPIV, RSV, CMV, PJ, and WU polyomavirus were more common in infants, while EBV was more common in preschool‐aged and school‐aged children. Li et al. conducted a retrospective analysis of 12,546 children with respiratory tract infections from 2018 to 2021 and reported that the incidence of bacterial infection was high in infants, the incidence of viral infection was high in preschool children, and atypical pathogens were the most common in children aged 3–5 years [22]. Zhang et al.’s study of 7668 patients with respiratory tract infections from 2019 to 2021 revealed that RSV, HRV, MP, and HPIV had relatively high detection rates in the infant group (0–6 months) and the young child group (7 months–2 years). The highest positive rates of respiratory pathogens in children (3–6 years) and adolescents (7–18 years) were associated with MP and HRV [21]. Lv et al.’s study of 4804 children with respiratory tract infections from 2019 to 2022 revealed that the infection rates of SP, RSV, and HPIV in the infant group were higher than those in other groups. The HRV infection rate was higher in young children than in other groups. The infection rates of Flu‐A, *Chlamydia pneumoniae*, MP, and HI in preschool‐aged and school‐aged children are higher than those in infants and young children [13].

Seasonal variation was also observed. In this study, more than half of the MP infections occurred in autumn. MTBC, HPIV, and CMV infections were more common in spring and summer. Flu‐B and ADV infections were more common in autumn and winter. RSV, Flu‐A, and *Fusobacterium nucleatum* infections were more common in winter and spring. *Enterovirus* infection was more common in summer and autumn. According to the Beijing Respiratory Pathogen Surveillance System, MP, HPIV, and HRV were the main pathogens identified in September 2023; MP, HPIV, and ADV were the main pathogens identified in October; and MP and RSV were the main pathogens identified in November. Other studies also revealed that MP infection was an important cause of pneumonia in autumn and winter of 2023, with an increase in the MP infection rate [25–29]. A study conducted by Shi and Huang on 10,396 children with acute respiratory infections from 2021 to 2022 revealed that ADV and *Chlamydia pneumoniae* infections occurred mostly in spring; that coronavirus and Flu‐A infections occurred mostly in summer; that HRV, HBoV, MP, and HPIV infections occurred mostly in autumn; and that RSV and Flu‐B infections occurred mostly in winter [19]. The seasonal prevalence of respiratory pathogens may be related to the following three factors: (i) humidity and temperature, (ii) human behavior (including indoor/outdoor activities, number of close contacts, etc.), and (iii) the effects of changing environmental conditions on host defense mechanisms [30, 31].

In this study, the high rates of MP and mixed infections, along with pathogen distribution differences by sex, age, and season, may be attributed not only to specimen and regional factors but also to post‐COVID‐19 immune dysregulation [32] and NPI‐induced “immune debt” [33].

This study has several limitations, including its single‐center design, short duration, and selection bias toward patients requiring bronchoscopy. In addition, this study did not analyze the association between pathogen distribution and clinical severity, prognosis, or treatment response. This limits the interpretation of the clinical significance of the detected pathogens. Therefore, the actual clinical value of the detected results requires further investigation. The study population consists of children with severe/complex CAP who underwent bronchoscopy; therefore, our findings may not be generalizable to mild or outpatient CAP cases. This selection bias is an inherent feature of the study design, and the interpretation of our results should be limited to this specific population. Moreover, the high detection rate in this study should be interpreted with caution. The high sensitivity of tNGS may lead to overdetection, and some low‐abundance organisms may represent colonization or background contamination rather than true causative pathogens. Therefore, the results should be integrated with clinical manifestations and laboratory findings for comprehensive interpretation.

In conclusion, this single‐center, retrospective study of 1526 pediatric CAP cases in 2023 reveals a distinct BALF pathogen profile, dominated by MP (99.9% overall detection, 62% mixed infections with EBV/HRV) and showing sex‐, age‐, and season‐specific distributions. Due to its design and lack of clinical correlation analysis, these findings should be interpreted as reflecting the local etiological landscape and may not be generalizable.

## Author Contributions

X.Z. and J.S. contributed equally to the manuscript. X.Z. and J.S. contributed to the conception and are responsible for the overall content. Z.W., X.Z., and J.S. designed the study. Z.W., Y.W., Y.C., T.H., and D.T. collected the data. Z.W., Y.G., M.W., X.Z., and J.S. analyzed and interpreted the data. Z.W., Y.H., Y.G., and M.W. contributed to the statistical analysis. Z.W. edited the manuscript. X.Z. and J.S. revised the manuscript.

## Funding

The authors have nothing to report.

## Disclosure

All authors approved the submitted and final versions. A preprint has previously been published [34].

## Ethics Statement

The study was approved by the Medical Ethics Review Committee of Maternal and Child Health Hospital of Hubei Province (No. 2024‐107‐01). All patients and their guardians gave informed consent.

## Consent

Please see the Ethics Statement.

## Conflicts of Interest

The authors declare no conflicts of interest.

## Data Availability

The data that support the findings of this study are available on request from the corresponding authors. The data are not publicly available due to privacy or ethical restrictions.
